# Psychotherapy in the Kurdistan region of Iraq (KRI): Preferences and expectations of the Kurdish host community, internally displaced- and Syrian refugee community

**DOI:** 10.1177/0020764021995219

**Published:** 2021-02-14

**Authors:** Kerem Böge, Eric Hahn, Judith Strasser, Stephanie Schweininger, Malek Bajbouj, Carine Karnouk

**Affiliations:** Department of Psychiatry and Psychotherapy, Charité – Universitätsmedizin, Campus Benjamin Franklin, Berlin, Germany

**Keywords:** Mental health, psychotherapy, Kurdistan, Iraq, Arab, refugees, asylum seekers, stigma, bias, satisfaction

## Abstract

**Background and Aim:**

The Kurdish Region of Iraq (KRI) is home to Kurds, internally displaced persons, and Syrian refugees. In the last decades, its inhabitants have witnessed a great deal of political instability, which has led to increased rates of psychological distress. Mental illness contrasts with limited access to and availability of mental health services – and so the treatment gap remains high. This study aims to investigate the perspectives, perceptions, and expectations of Syrian refugees, internally displaced persons and KRI host community members concerning mental health care in the governorate of Duhok. Attitudes and perspectives regarding psychotherapy, such as satisfaction with services, effects of therapy, bias toward therapy, and stigma, are explored.

**Methods:**

One hundred one participants were recruited from hospitals, clinical settings, and institutions from the governorate of Duhok in the KRI. Participants received the Patient Satisfaction Questionnaire (PSQ) and were asked to evaluate services through four subscales: patient satisfaction, effects of therapy, bias toward therapy, and stigma.

**Results:**

Results revealed overall high satisfaction with services and effects of therapy. In contrast, both bias and stigma subscales were rated more ambivalently.

**Conclusion:**

Patient satisfaction is key for assessing health care quality, understanding attitudes toward therapy, and help-seeking behavior. Results offer insight for stakeholders in the psychosocial field allowing for a better understanding and improvement of availability and access to quality-driven mental health care services

## Introduction

In recent years, rising numbers have revealed that there are more displaced persons as a result of events following the Arab Spring than those reported after the second World War (Cetorelli et al., 2017; Ibrahim & Hassan, 2017; Okasha et al., 2012). Ongoing conflicts and political unrest in the region have forced millions to seek refuge in neighboring countries (Lebanon, Turkey, Jordan, Iraq, and the semi-autonomous Kurdish region of Iraq) (Ibrahim & Hassan, 2017), and afar. Together, both host and refugee communities must bear the high cost of war – material and non-material (Cetorelli et al., 2017). According to Fasfous et al. (2013), as a result of ongoing conflict, most individuals in Middle Eastern conflict zones have been exposed to at least one traumatic experience in their lifetime. These events have led to a widely documented increase in rates of psychological distress and trauma (Ibrahim & Hassan, 2017) – all of which are well known risk factors for the development of mental illness (Kurdistan Regional Statistics Office [KRSO], International Organization for Migration [IOM] & the United Nations Population Fund [UNPF], 2018). Although host governments are working closely with local and international organizations to adequately meet the physical and mental health needs of these communities, the treatment gap remains high and sustainable solutions are scarce (Bolton, 2013; Cetorelli et al., 2017).

Iraq is a predominantly Muslim Arab country with over 30 million inhabitants, who have witnessed a considerable amount of war, sectarian violence, and political turmoil in the last decades (Sadik et al., 2010). Its unique geographical location, diverse population, and profound contemporary history (2003 Iraq War up to now) lays ground for its complex ongoing political struggles (Sadik et al., 2010). Within Iraq, the Kurdistan Region of Iraq (KRI) has a population size of about five million inhabitants, spread across three main governorates: Sulaymaniyah, Erbil, and Duhok (KRSO, IOM & UNFPA, 2018). According to an official census by the United Nations High Commissioner of Refugees (UNHCR and REACH Initiative, 2015), about 226,934 Syrian refugees had fled to KRI, mostly spread across nine camps in the region (KRSO, IOM & UNFPA, 2018). Among those seeking refuge are also minority groups, such as the Yazidis, who have suffered a great deal of persecution and marginalization from previous regimes and now religious extremists (Cetorelli et al., 2017). According to a study by Ibrahim and Hassan (2017), most refugees in the KRI region reported to have fled for two reasons: (1) a general feeling of safety and stability within the region and (2) a familiar language, nationality, as well as transferable professional skills to the host community (Ibrahim & Hassan, 2017; Salman, 2012).

Historically, wars and conflict-settings have been found to contribute negatively to both mental and physical well-being (Ibrahim & Hassan, 2017). In 2014, an analysis which reviewed nine studies in the Arab region showed a significant effect of war trauma on overall psychological health (Al-ghzawi et al., 2014), with the highest incidence of distress reported by internally displaced people (IDP), who are temporarily located in camps in the KRI region (Cetorelli et al., 2017). Although there are no recent official statistics on the exact prevalence rates of mental illness in the KRI, according to Bolton (2013), the Iraq Mental Health Survey of 2007 revealed an ‘increasing lifetime prevalence of most disorders across generations’. So far, the most pronounced disorders in both host and refugee communities, have been PTSD and depression among other disorders (Bolton, 2013; Ibrahim & Hassan, 2017; Naja et al., 2016). Furthermore, mental health treatment in Iraq is scarce, centralized, urbanized and relatively recent, with its first initiatives starting in the late 1970s (Al-Salihy & Rahim, 2013). While some sources report one psychiatrist per 300,000 inhabitants before the year of 2003 (Sadik et al., 2010), others estimate that there are fewer than 1,000 psychiatrists in all of Iraq – most of whom are located in hospital settings, do not offer therapy due to time constraints and rely heavily on prescribing medication (Bolton, 2013).

Although there is currently a rapid transformation in the health system and efforts from international key players and local counterparts alike to offer better psychiatric care, several challenges are still present (Aziz et al., 2014), such as limited training, mental health education, and the absence of formal and official evaluations of the existing psychiatric services in the KRI (Al-Salihy & Rahim, 2013). Additionally, the mental health system in the KRI is heavily dominated by bureaucratic and hierarchical systems (Al-Salihy & Rahim, 2013), making it harder to allocate services where they are actually needed. In a study by Aziz et al. (2014), it was found that Syrian refugees living in the KRI generally had scored high on social relationships, indicating a good level of social support, but had lower scores on domains related to physical and mental health. The paper further urges future research and mental health initiatives to prioritize physical and psychological health for the improved well-being of refugees in the KRI region. Without the support of evidence that can bring light to the current state of affairs regarding the diverse psychological needs of both the host and refugee communities within the KRI, the accurate allocation of funds and resources in the right places will not be possible.

Even though Iraq does not have an official mental health policy, over the years, some ministries, including those of KRI, have acknowledged the treatment gap and are working toward finding solutions (Bolton, 2013). Not only are services scarce and inaccessible, local governments and organizations are also facing cultural and social challenges related to negative attitudes and biases toward mental illness and help-seeking behaviors in the KRI region (Bolton, 2013). Stigma has been known to be one of the leading barriers to seeking treatment in the Arab world (Okasha et al., 2012; Sadik et al., 2010; Westbrook et al., 1993). According to a needs assessment by John Hopkin’s School of Public Health, mental health-related stigma in Iraq is higher than in other parts of the world (Bolton, 2013) and Iraqis are often reluctant to seek treatment due to a fear of familial and social marginalization. According to Sadik et al. (2010), in a population-based survey, covering five Baghdadi districts, about attitudes toward mental illness, most respondents saw mental illness as a weakness, were ashamed of it and gave mixed opinions concerning the relationship between psychological distress, work, and marriage. Similarly, another study investigating host and refugee-community members’ perspectives on psychotherapy showed overall high rates of satisfaction with provided services, but an ambivalence regards stigma and bias toward therapy (Karnouk et al., 2019). Despite these challenges and barriers, the growing body of research seems to be having an impact on governmental policies, community-based initiatives and increasing access to mental health services (Cetorelli et al., 2017). Not only are investigations of existing-services necessary, but also a key predictor in offering more suitable, effective and culturally-sensitive treatment options.

Within this context, this study contributes to a dearth of available literature and offers a unique glimpse into the perspectives, perceptions, and expectations of Syrian refugees, internally displaced persons (IDPs) and KRI host community members concerning mental health care in the governorate of Duhok. Other public experiences regarding psychotherapy, such as satisfaction with services, effects of therapy, bias toward therapy and stigma, will also be explored.

## Methods

### Participants

A sample of 101 patients was recruited between October and December 2017 in the Kurdish Region of Iraq (KRI). All participants in the current study received psychotherapy- or counseling sessions at various organizations specialized in offering mental health services. Structured interviews assessing sociodemographic information and a self-report measure (PSQ) were administered by local- as well as international interviewers in Arabic, Sorani or Kurmanji; depending on the location of the beneficiaries and their background. The interviews were conducted by psychologists or psychiatrists and were not linked to the service provision. In cases of illiteracy, the interviewers noted the responses for participants and provided clarification on the items when necessary. All interviewers received a thorough and in-depth structured training of 2 days in the form of a workshop in order to guarantee consistency in the interview process.

*Inclusion criteria* were defined as (a) age between 18 and 75 years; (b) belonging either to the Kurdish host-, internally displaced-, or Syrian refugee community; (c) obtained counseling- or psychotherapy sessions within the last 6 months, (d) attended > 4 sessions prior assessment, (e) receiving pharmacological treatment was permitted.

### Procedure

The recruitment of a representative sample was not feasible since younger and female individuals were mostly seeking treatment by our local partners and interested in participation. Therefore, the study design aimed to balance the three patient groups according to their socio-demographic variables, including gender and background (host-, the Syrian refugee community and internally displaced people [IDP]).

In the KRI cooperating local and international organizations recruited suitable candidates by asking for their willingness to participate in the study. Potential participants who met the inclusion criteria received a study information sheet, were encouraged to ask any questions that remained unclear and upon agreement signed an informed consent. The sample was recruited from the following organizations: Azadi Teaching Hospital, Child and Adolescent Mental Health Center Duhok, Emma Organization, Erbil Psychiatric Hospital, International Medical Corps, International Organization for Migration, Jiyan Foundation for Human Rights, Koya University, Mercy Corps, SEED Foundation, Survivor Center Duhok, Terre des Hommes Italy, Wchan, and World Vision International in the Kurdish region of Iraq.

Matching gender between interviewers and participants could not always be ensured in the study due to structural, logistic and personnel challenges. No financial compensation was offered to the participants besides travel costs. However, all participants received a telephone hotline number allowing for follow-up psychological support if needed. Subsequent to data assessment of the pencil-paper questionnaires, all data was translated by local translators into English. Finally, data were entered into a Statistical Package for Social Science (SPSS) spreadsheet and electronically saved. The ethical committees of Charité – Universitätsmedizin Berlin, Germany accepted the study design in accordance with the latest version of the Declaration of Helsinki.

### Assessment

Culturally sensitive and adequate questionnaires assessing patient perceptions and preferences concerning psychotherapy- and counseling sessions remain scarce in the Arab world. To address this need, an instrument was designed in close collaboration with the NGO Misereor.

The Patient Satisfaction Questionnaire (PSQ) was specifically developed to evaluate patient needs and perceptions concerning relevant psychotherapeutic processes in the MENA region. The instrument has also been successfully used in similar regions, including the Kingdom of Jordan and showed its clinical utility by our research group (see Karnouk et al., 2019). The items are partially based on the well-known PSQ measure by Ware et al. (1983), however, some items were adapted by Miseror and our research team, in order to ensure applicability in the MENA region. These include perceived bias, effects of the therapy, stigma, and patients’ satisfaction, containing dimensions, such as beliefs, perceptions, and expectations. The PSQ is a self-report questionnaire originally developed in Arabic containing four subscales covering the main broad domains of mental health care provision. In total, the scale consists of 26 items. Furthermore, it is divided into four subscales with varying item distribution: patient satisfaction (9), bias (6), effects of therapy (7), and stigma (4). Responses for each of the subscales’ items are scored on a 5-point Likert scale with diverse anchor points (details stated in Tables 2–5). For the current study, inconsistent to excellent consistency was found for the four subscales with Cronbach’s alpha ranging from α_Satisfaction_ = .897, α_Bias_ = .419, α_EffectsofTherapy_ = .880, α_Stigma_ = .705 (Tavakol & Dennick, 2011).

### Statistical analysis

Prior to the analysis, assumptions of normality (values of skewness and kurtosis), outliers and sphericity were assessed. In the first step, descriptive and inferential statistics for the Patient Satisfaction Questionnaire (PSQ) were calculated. Next, the central tendency of continuous measures was calculated and displayed by frequencies, percentages, means, standard deviations and range of variables. For all categorical variables and subscale items, percentages and actual counts are presented to illustrate missing measures. To examine the possible difference between three patient groups (host-, the Syrian refugee community, and internally displaced people [IDP]), subsample analyses will be performed using non-parametric Kruskal–Wallis one-way analysis of variance tests. All collected data was collected and stored in a spreadsheet using the Statistical Package for the Social Science (SPSS) 25, MacOS-X. Statistical analyses will be set at an exploratory significance level of *p* < .5.

## Results

A total of 104 participants were analyzed in the present study. 61.5% were female, while age ranged from 18 to 74, with a mean of 35.04 (SD = 11.64). The majority of participants were Kurdish (86.1%) and indicated Islam as their religion (69.3%). Nearly half of the total sample was from the host community (47.9%), while 22.3% were from Syria (Syrian refugee community), and 29.8% were from the internally displayed community (IDP). All assessed sociodemographic variables are shown in detail in Table 1. Moreover, all descriptive results of frequencies, percentages, means, standard deviations, and range of variables are shown in Tables 2 to 5. Each of the four subscales’ mean and test results is depicted in Table 6. Non-parametric Kruskal–Wallis one-way of variance test were conducted demonstrating no significant difference between all four subscales across the three subsamples: χ^2^_Satisfaction_ (2) = 0.126, *p* = .939; χ^2^_Bias_ (2) = 1.478, *p* = .478; χ^2^_EffectsofTherapy_ (2) = 4.663, *p* = .097; χ^2^_Stigma_ (2) = .304, *p* = .859.

**Table 1.** Sociodemographic characteristics of the survey sample.

Sociodemographic data	*N* = 104
Gender (%, *n* = 97)	
Male	33.7
Female	61.5
Age in years (%, *n* = 100)	35.04 (11.64)*
18– 28	34.0
29– 39	34.0
40– 54	25.0
55– 74	7.0
Ethnicity (%)	
Arab	12.9
Kurdish	86.1
Turkmen	1.0
Religion (%)	
Muslim	69.3
Yazidi	30.7
Target group (%, *n* = 94)	
Host community	47.9
Syrian refugee community	22.3
IDP community	29.8
Waiting time (%, *n* = 100)	
Same day	69.0
1–7 days	22.0
1– 2 weeks	5.0
3– 4 weeks	1.0
More than 4 weeks	3.0

*Note.* Waiting time = waiting time between registration with the organization and first contact with a physician or psychologist, IDP = internally displaced people.

* Mean and standard deviation for age.

**Table 2.** Descriptive statistics of the Patient Satisfaction Questionnaire.

How was the doctor or nurse at. . .	Very poor (%)	Poor (%)	Fair (%)	Good (%)	Very good (%)	Mean (SD)
Making you feel at ease?	0	3.0	11.9	21.8	63.4	4.46 (0.82)
Letting you tell your story?	1.0	1.9	5.8	27.9	63.5	4.51 (0.78)
Really listening? (*n* = 103)	0	2.9	3.9	28.2	65.0	4.55 (0.71)
Being interested in you as a whole person? (*n* = 103)	0	2.9	4.9	35.0	57.3	4.47 (0.73)
Fully understanding your concerns? (*n* = 103)	0	1.9	4.9	26.2	67.0	4.58 (0.68)
Showing care and compassion?	1.9	3.8	7.7	21.2	65.4	4.44 (0.93)
Explaining things clearly? (*n* = 103)	1.0	1.9	4.9	35.0	57.3	4.46 (0.76)
Helping you to take control?	1.9	1.9	7.7	32.7	55.8	4.38 (0.86)
Overall rating of the consultation / treatment	2.9	1.0	8.7	24.0	63.5	4.44 (0.91)
Overall rating on the Patient Satisfaction Questionnaire	1.0	2.3	6.7	27.9	62.0	4.47 (0.79)

*Note.* The full sample consisted of *N* = 104 participants. Very poor = 1, Poor = 2, Fair = 3, Good = 4, Very good = 5.

**Table 3.** Descriptive statistics of the Bias Questionnaire.

It is acceptable if the therapist. . .	Totally disagree (%)	Somewhat disagree (%)	Undecided (%)	Somewhat agree (%)	Totally agree (%)	Mean (SD)
Is a man. (*n* = 103)	48.5	10.7	7.8	5.8	27.2	2.52 (1.73)
Is a woman. (*n* = 101)	74.3	4.0	5.0	7.9	8.9	1.73 (1.36)
Has a different opinion regarding national politics. (*n* = 99)	16.2	2.0	4.0	18.2	59.6	4.03 (1.48)
Is from another country. (*n* = 101)	63.4	3.0	10.9	10.9	11.9	2.05 (1.51)
Belongs to a different ethnic group. (*n* = 100)	50.0	10.0	11.0	7.0	22.0	2.41 (1.65)
Belongs to a different religious group. (*n* = 102)	64.7	5.9	3.9	6.9	18.6	2.09 (1.63)
Overall rating on the Bias Questionnaire	53.0	5.9	7.1	9.4	24.6	2.46 (1.56)

*Note.* The full sample consisted of *N* = 104 participants. Totally disagree = 1, Somewhat disagree = 2, Neither agree nor disagree = 3, Somewhat agree = 4, Totally agree = 5.

**Table 4.** Descriptive statistics of the Effects of the Therapy Questionnaire.

	Totally disagree (%)	Somewhat disagree (%)	Undecided (%)	Somewhat agree (%)	Totally agree (%)	Mean (SD)
I mostly feel relieved after the therapy sessions.	1.9	4.8	8.7	23.1	61.5	4.38 (0.97)
The therapy helped me to handle my problems and my distress. (*n* = 103)	1.9	4.9	7.8	35.9	49.5	4.26 (0.94)
Now I can understand much better, where my problems came from. (*n* = 101)	4.0	5.9	8.9	22.8	58.4	4.26 (1.10)
With therapy it is easier for me to face the difficulties in my life. (*n* = 103)	1.9	3.9	11.7	34.0	48.5	4.23 (0.94)
Therapy gave me new hope and new perspectives for my life. (*n* = 103)	1.0	5.8	10.7	29.1	53.4	4.28 (0.64)
I now get along better with the people in my immediate environment. (*n* = 102)	2.0	3.9	6.9	38.2	49.0	4.28 (0.91)
Therapy helped me to find solutions for my problems.	2.9	3.8	12.5	33.7	47.1	4.18 (0.99)
Overall rating on the Effects of the Therapy Questionnaire	2.2	4.7	9.6	31.0	52.5	4.27 (0.93)

*Note.* The full sample consisted of *N* = 104 participants. Totally disagree = 1, Somewhat disagree = 2, Neither agree nor disagree = 3, Somewhat agree = 4, Totally agree = 5.

**Table 5.** Descriptive statistics of the Stigma Questionnaire.

	Totally disagree (%)	Somewhat disagree (%)	Undecided (%)	Somewhat agree (%)	Totally agree (%)	Mean (SD)
I think if others know about my psychological problems, they lose respect for me.	52.9	13.5	12.5	10.6	10.6	2.12 (1.43)
I am afraid of possible disadvantages in regard to my family planning and family life because of my psychological problems.	23.1	12.5	14.4	26.0	24.0	3.15 (1.51)
I am scared that people are thinking or talking about me in a negative way because I am in therapy for my psychological problems. (*n* = 103)	47.1	12.5	8.7	19.2	12.5	2.38 (1.53)
I feel ashamed that I have to go to a therapist for my problems. (*n* = 103)	65.0	10.7	5.8	11.7	6.8	1.84 (1.33)
Overall rating on the Stigma Questionnaire	47.0	12.3	10.4	16.9	13.5	2.37 (1.45)

*Note.* The full sample consisted of *N* = 104 participants. Totally disagree = 1, Somewhat disagree = 2, Neither agree nor disagree = 3, Somewhat agree = 4, Totally agree = 5.

**Table 6.** Descriptive statistics and analysis of subsample differences for all subscales (Kruskal–Wallis one-way analysis of variance test).

Outcome variable	Mean rank	*p*
Satisfaction subscale		
Host community (*n* = 45)	47.17	.939
Syrian refugee community (*n* = 21)	47.76	
IDP community (*n* = 29)	49.47	
Bias subscale		
Host community (*n* = 45)	50.21	.478
Syrian refugee community (*n =* 21)	41.64	
IDP community (*n =* 29)	49.17	
Effects of the therapy subscale		
Host community (*n =* 45)	52.21	.097
Syrian refugee community (*n =* 21)	51.62	
IDP community (*n =* 29)	38.84	
Stigma subscale		
Host community (*n =* 45)	46.57	.859
Syrian refugee community (*n =* 21)	50.50	
IDP community (*n =* 29)	48.41	

*Note.* IDP = internally displaced people.

### Patient satisfaction

In total, results indicate very high satisfaction rates on all eight items of the subscale with a mean of 4.47 (SD *=* 0.79). Sixty-two percent rated the interpersonal level of treatment with ‘very good’ and 27.9% with ‘good’. Therewith, only 2.3 ranked treatment provision with ‘poor’ and 1% with ‘very poor’ – 6.7% stated ‘fair’. All responses on the satisfaction subscale are displayed on an item and overall level with frequencies, means and standard deviations in Table 2.

### Bias

Bias in the form of attitudes toward the treatment provider was rated on six items. Participants showed moderate levels of bias, with an average response of 2.45 (SD *=* 1.56). A majority of 58.9% showed reservations regarding gender, religious affiliations, ethnicity and country of origin with their therapist – 53% ‘totally disagree’ and 5.9% ‘somewhat disagree’. Only 34% accepted possible differences with 9.4% ‘somewhat agree’ and 24.6% ‘totally agree’, respectively. A minority of 7.1% remained undecided.

Table 3 shows all descriptive statistics on an overall and item level for the bias subscale.

### Effects of therapy

Overall, approval rates for the effects of therapy were very high, with 4.27 (SD *=* 0.79) on average. About 83.5% were endorsing the treatment effects, split to 52.5% responding with ‘totally agree’ and 31% with ‘somewhat agree’. Less than 7% did not find the treatment effects favorable with 2.2% rating it as ‘totally disagree’, and 4.7% as ‘somewhat disagree’. Roughly, 9.6% of all participants remained undecided. Responses for the subscale effects of therapy and its seven items are presented in Table 4.

### Stigma

For the stigma subscale, all four items are phrased reversed; lower rates of approval display, therefore, lower levels of stigma. For the current sample, the mean score was at 2.37 (SD = 1.45), depicting moderate to low levels of stigma. While a majority of 47.0% rejected self-stigmatizing items (‘totally disagree’), answer patterns across all other anchors were distributed equally with 12.3% ‘somewhat disagree’, 10.4% ‘undecided’, and 16.9% ‘somewhat agree’ and 13.5% ‘totally agree’, respectively. Table 5 illustrates all descriptive statistics for the bias subscale with its four items.

## Discussion

Result of the present study indicate higher rates of acceptance concerning psychological services among participants in the KRI region, contrasting with previous research revealing a reluctance to engage in psychiatric treatment from the side of both the patient and their families in Iraq, particularly for women (Bolton, 2013). The main study findings reveal high levels of satisfaction with psychological interventions, an overall positive evaluation of the effects of therapy, low to moderate levels of perceived public stigma and moderate levels of biases related to therapist characteristics. Several logistical and attitudinal factors may have contributed to these positive changes ranging from an increased need for services to growing public and organizational efforts leading to increased availability, access to and quality of mental health services.

Both subscales ‘patient satisfaction’ and ‘effects of therapy’ were rated positively. Participants reported being relatively satisfied with available services, particularly high scores were given in items such as, feeling heard, understood, with and grasping things more clearly. Moreover, participants also rated the effects of therapy as high, with the most pronounced items being: feeling relieved after sessions, having fresh perspectives and coping better in general. In a similar study in Jordan by Karnouk et al. (2019), respondents who were mostly women and had a similar age group, also rated levels of satisfaction with services as high. Nevertheless, it is worth mentioning that short waiting periods may also have an impact on higher positive ratings and satisfaction with services – a criterion commonly cited as a measure for good quality standards in mental health care settings (Hasler et al., 2004).

In contrast to high levels of satisfaction, stigma and biases were rated more ambivalently. Participant ratings for the stigma subscale ranged between low to moderate. Concerns related to how others would view the individuals if they sought treatment have long been a barrier to seeking psychological care (Pedersen & Paves, 2014). Other studies in the Arab region have reported similar or even higher results (Nasir & Al-Qutob, 2004; Okasha et al., 2012). Furthermore, the study reveals an apparent dissonance between, on the one hand, having an open and positive attitude toward therapy and on the other, having a negative perceived public stigma related to it. There is a growing need to provide mental health services in the Middle East (Böge et al., 2020; Bolton, 2013). In recent years, efforts for increasing the availability of and access to mental health care services in the KRI region have increased. According to Henderson et al. (2013), increased use of services and treatment-seeking behaviors lead to lower rates of public stigma. As opposed to our study results, existing literature points toward stigma being relatively higher in Iraq when comparing to other places (Bolton, 2013). However, in a study by Petty et al. (2006), participants with changing attitudes evaluated matters more neutrally than their initial attitude. In this study, participants reported having no shameful feelings associated with therapy and did not worry that others would lose respect for them. However, one dominant consensus was with regard to fears related to family planning. The family unit is known to be a powerful pillar in Arab culture, especially for women, who make up most of our sample. In another study by Sadik et al. (2010), most participants saw mental illness as a ‘weakness’ and had concerns regarding the effect of their psychological distress on marriage prospects (Awad et al., 2013; Heath et al., 2016).

Furthermore, the ‘bias’ subscale was rated ambivalently with some contradictory items standing out, particularly with regards to specific characteristics related to the therapist, such as gender and political opinion (Karnouk et al., 2019). Whereas more than half of the sample did not find it acceptable if the therapist ‘is a man’, 74% did not find it acceptable for the therapist to be ‘a woman’ either. Given that most of the respondents in this study are females, these findings demonstrate an interesting contradiction. In the literature, gender biases have been reported to be particularly strong in predominantly Muslim countries, with previous research reporting clear preferences and openness to therapists who are women, particularly for female clients for reasons related to cultural norms and gender roles (Heath et al., 2016). Moreover, this paradox may also be a feature of attitude change and openness (Petty et al., 2006). Nonetheless, it is still unclear whether these results may be due to vague wording/phrasing in the questionnaire. It makes it therefore challenging to understand whether these attitudes stem from cultural norms and gender roles, or whether they truly reflect the respondents preferences in matching genders. For future use, it would be beneficial for this item to be reviewed and for the overall Patient Satisfaction Questionnaire to be refined and validated in order to improve its accuracy and usefulness in the region, where assessments are often scarce.

Furthermore, respondents mentioned having no biases with regards to a difference in national politics between therapist and client. However, the therapist being of the same nationality, ethnic and/or religious group was clearly preferred. Factors such as trust, perceived stigma and a fear of the therapist not connecting to the client’s reality may be playing a role here, especially that our sample includes several minorities, such as Kurds and Yazidis. The sample characteristics are culturally diverse, capturing the real-life setting and demonstrating the ‘ecological validity’ of our study.

Several strengths and limitations were identified in this exploratory study. Although the sample was recruited through different hospitals and organizations within governate of Duhok, the sample was not randomized, and it is most likely that only participants who showed interest and a willingness to take part in the study were recruited. Therefore, it may be possible that because the this was a convenience sample, patients with lower satisfaction may not have taken part in the study, contributing to inflated levels of satisfaction with therapy and mostly positive reports with regards to its effects. Although the analysis revealed no subsample differences between the different community members, it would be interesting to conduct a replication or confirmatory study with larger sample size. Moreover, the KRI is a diverse region, with differences in socio-political structures, religious orientations, ethnicity and more. Due to a scarcity in time and availability of resources, the collections of valuable information and ‘qualitative’ experiences of respondents were not possible at this point. Furthermore, relevant information with regards to the length of overall treatment, type of therapy, and/or diagnoses would have been interesting to analyze. It would also be useful to build on these findings

In conclusion, the study provides evidence for the growing acceptance of mental health services and positive changes toward accessing and receiving psychotherapy in the KRI. Understanding patient satisfaction and the effects of therapy are essential indicators for improving access to and the quality of mental health services. These investigations allow for more practical plans and an accurate allocation of resources. In turn, positive experiences will cause a decrease in stigma and biases. While the treatment gap remains high in KRI, governmental- and other developmental efforts are collaboratively tackling mental health-related issues and aiming to improve mental health care services, therefore investigating client preferences and expectations and examining their quality can also improve existing infrastructures and create a system whereby both therapist and client can benefit from treatment conditions. Within that context, follow-up studies capturing the effects of these efforts on decreasing rates of psychological distress would be valuable sources of information that are useful for refugees, host community members, IDP’s and service providers alike.
